# Predicting microvascular invasion in small (≤ 5 cm) hepatocellular carcinomas using radiomics-based peritumoral analysis

**DOI:** 10.1186/s13244-024-01649-0

**Published:** 2024-03-26

**Authors:** Fang Wang, Ming Cheng, Binbin Du, Jing Li, Liming Li, Wenpeng Huang, Jianbo Gao

**Affiliations:** 1 Department of Radiology, The First Affiliated Hospital of Zhengzhou University, 1 Jianshe East Road, Erqi, Zhengzhou, Henan 450052 People’s Republic of China; 2 Information Department, The First Affiliated Hospital of Zhengzhou University, Zhengzhou, People’s Republic of China; 3 Vasculocardiology Department, The First Affiliated Hospital of Zhengzhou University, Zhengzhou, People’s Republic of China; 4 Department of Radiology, The Affiliated Cancer Hospital of Zhengzhou University, Zhengzhou, People’s Republic of China

**Keywords:** Carcinoma, Hepatocellular, Microvascular invasion, Radiomics, Tomography (X-ray computed)

## Abstract

**Objective:**

We assessed the predictive capacity of computed tomography (CT)-enhanced radiomics models in determining microvascular invasion (MVI) for isolated hepatocellular carcinoma (HCC) ≤ 5 cm within peritumoral margins of 5 and 10 mm.

**Methods:**

Radiomics software was used for feature extraction. We used the least absolute shrinkage and selection operator (LASSO) algorithm to establish an effective model to predict patients’ preoperative MVI status.

**Results:**

The area under the curve (AUC) values in the validation sets for the 5- and 10-mm radiomics models concerning arterial tumors were 0.759 and 0.637, respectively. In the portal vein phase, they were 0.626 and 0.693, respectively. Additionally, the combined radiomics model for arterial tumors and the peritumoral 5-mm margin had an AUC value of 0.820. The decision curve showed that the combined tumor and peritumoral radiomics model exhibited a somewhat superior benefit compared to the traditional model, while the fusion model demonstrated an even greater advantage, indicating its significant potential in clinical application.

**Conclusion:**

The 5-mm peritumoral arterial model had superior accuracy and sensitivity in predicting MVI. Moreover, the combined tumor and peritumoral radiomics model outperformed both the individual tumor and peritumoral radiomics models. The most effective combination was the arterial phase tumor and peritumor 5-mm margin combination. Using a fusion model that integrates tumor and peritumoral radiomics and clinical data can aid in the preoperative diagnosis of the MVI of isolated HCC ≤ 5 cm, indicating considerable practical value.

**Critical relevance statement:**

The radiomics model including a 5-mm peritumoral expansion is a promising noninvasive biomarker for preoperatively predicting microvascular invasion in patients diagnosed with a solitary HCC ≤ 5 cm.

**Key points:**

• Radiomics features extracted at a 5-mm distance from the tumor could better predict hepatocellular carcinoma microvascular invasion.

• Peritumoral radiomics can be used to capture tumor heterogeneity and predict microvascular invasion.

• This radiomics model stands as a promising noninvasive biomarker for preoperatively predicting MVI in individuals.

**Graphical Abstract:**

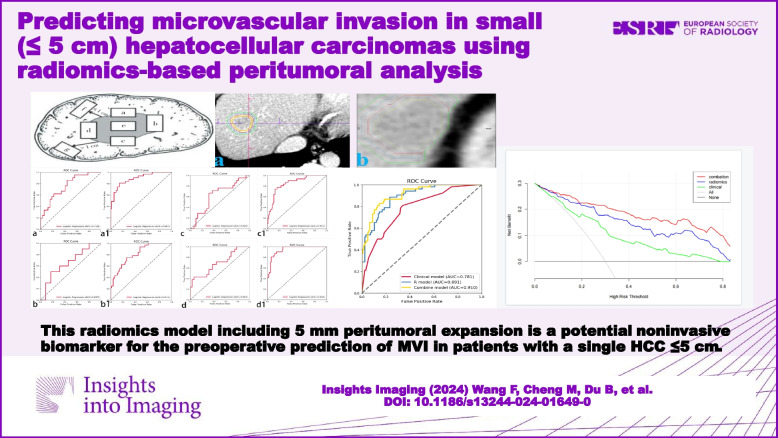

## Introduction

According to survey data released by the International Center for Research on Cancer, primary liver cancers rank as the sixth most prevalent malignant tumors globally, with the third highest mortality rate [1]. The rapid development of medical technology, particularly the advancement of examination technology, has improved the diagnosis and prognosis for individuals afflicted with hepatocellular carcinoma (HCC), resulting in improving trends in morbidity and mortality [2, 3]; however, the prognosis and long-term survival of HCC patients are still not optimistic [4, 5].

Microvascular invasion (MVI) typically occurs in the small branches of the portal veins of precancerous tissues, with occurrences in the hepatic vein branches being uncommon. The Milan criteria are internationally recognized benchmarks used to assess a patient’s suitability for liver transplantation. Among those with early-stage HCC who meet these benchmarks, the 5-year survival rate post-liver transplantation is approximately 75%, making this an efficient use of organ resources [6]. Research shows that MVI is an independent prognostic factor that impacts the outcomes of patients with HCC [7–10]. In addition, comparative analysis with pathological results has shown that mononodular tumors with outward protrusion and polynodular fusion are more prone to MVI [11]. However, the confirmation of imaging features depends on experienced experts, who may view certain features differently; therefore, the clinical use of subjective image interpretations is limited.

Radiomics involves extracting subtle features from macroscopic images, revealing microscopic heterogeneity within tumors [12]. Li et al. extracted and quantified features from magnetic resonance imaging (MRI) images of breast cancer and found that large tumors were more aggressive and had greater internal heterogeneity [13]. Some scholars conducting feature extraction on MRI images of patients with HCC found that radiomics features could effectively aid in classifying and predicting both the pathological grade of HCC and the presence of MVI [14, 15]. Kim et al. found that their model, including both the tumor and the 3- and 5-mm peritumoral margins, had higher predictive efficiency compared to the simple tumor feature model [16].

There is a significant difference in biological behavior when the tumor is larger than 5 cm. The AJCC-UICC TNM stage 8 designates this threshold as the boundary for T2 classification. Despite many reports on radiomics to predict MVI in HCC using computed tomography (CT) and MRI images, there is a noticeable lack of attention given to this specific subgroup of tumors. Therefore, isolated HCC ≤ 5 cm were the subject of this study, and tumor and peritumoral histological characteristics were extracted to explore and establish a preoperative model capable of accurately predicting the MVI status of HCC before surgery, offering valuable guidance for precision clinical treatment decisions.

## Methods

This retrospective study received approval from the ethics committees of the local institutions. Enrolled patients and their families were not required to provide informed consent.

### Patient selection

We retrospectively analyzed 206 patients diagnosed with isolated HCC (≤ 5 cm) at our hospital from 2017 to 2021. Among them were 177 men and 29 women. The inclusion criteria comprised (1) the presence of a solitary HCC with a maximum diameter of ≤ 5 cm, (2) lack of extrahepatic metastasis or major vascular invasion per preoperative imaging, (3) no previous HCC-related treatments, (4) comprehensive histopathologic descriptions of HCC, and (5) preoperative enhanced CT with adequate image quality performed within 1 week preceding the surgery.

The exclusion criteria comprised (1) incomplete pathological information, (2) tumors exhibiting diffuse growth and indeterminable edges, (3) concurrent existence of additional malignant tumors, and (4) inadequate clinical baseline data.

The external validation group included 60 patients from another hospital who met the inclusion criteria.

We used SPSS software to randomly split the study population into the training and validation groups. The enrolled patients’ clinical baseline data were obtained by accessing electronic medical records.

### CT imaging protocol

The CT scanning equipment used in this study included the Discovery 750 HD CT (GE Medical, USA), the SOMATOM Force CT (Siemens Medical, Germany), the Brilliance ICT (PHILIPS, The Netherlands), and the Aquilian One 640 (Toshiba, Japan).

### Patient scanning position

A 120-kV tube voltage with automatic tube current technology was used. The contrast agent (370 mg/mL) was injected via the right anterior elbow vein using a high-pressure syringe (rate = 3 mL/s; dose = 1.5 mL/kg). A rinse of physiological saline followed (20 mL). Using the small-dose trigger technique, imaging was initiated when the descending aorta reached 100 HU post-contrast agent injection. The arterial phase images were captured 10 s afterward, while the portal vein phase images were captured 30 s afterward.

### Radiomics feature analysis

#### Image segmentation and preprocessing

**Fig. 1:**
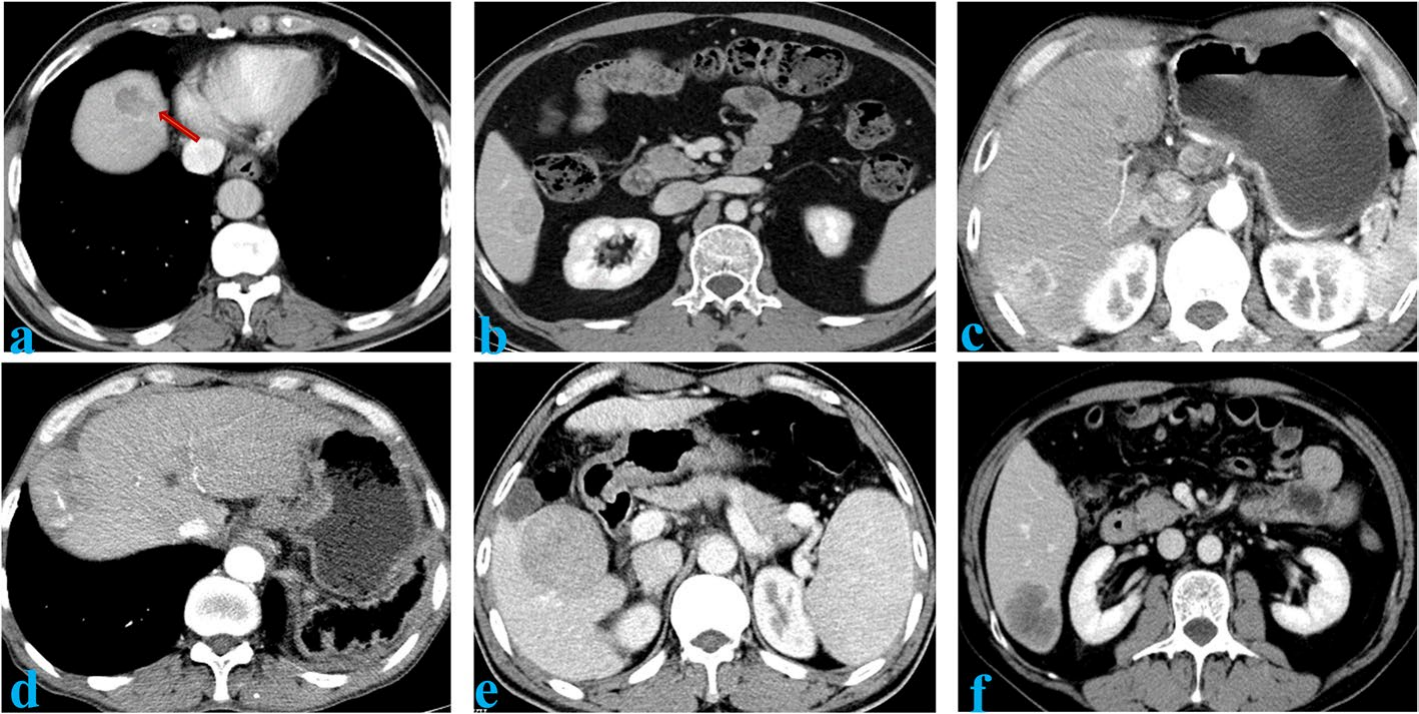
Image features: **a** Smooth edge of the tumor. **b** Complete capsule. **c** Peritumor enhancement. **d** Discontinuous intratumoral artery. **e** Peritumoral low-density ring. **f** Clear differences at the liver-tumor interface

The images obtained by different machines were reconstructed with the standard algorithm. The slice thickness and the layer spacing were 1 mm each. The window width was adjusted to 220 HU, while the window position was calibrated to 40 HU. In the absence of pathological results, the features of lesions on the images were assessed by two liver disease imaging specialists (one with 10 years of experience and the other with 15). When the two radiologists disagreed on certain features, a third physician with 20 years of experience double-checked the features. As shown in Fig. 1, the imaging features included the following: (1) tumor size; (2) tumor margin (smooth or rough); (3) capsule (no capsule, complete capsule, or incomplete capsule); (4) peritumoral enhancement; (5) intratumoral artery; (6) peritumoral low-density rim; and (7) liver-tumor interface differences.

#### Traditional diagnostic imaging features of MVI

RVI (radiogenomic venous invasion) was characterized by the existence of intratumoral arteries without the presence of a low-density rim and without notable differences in the liver-tumor interface [17]. TTPVI (two-trait predictor of venous invasion) was described as the occurrence of intratumoral arteries and the lack of a low-density rim around the tumor [18].

#### Delineated volume area of interest (VOI)

**Fig. 2:**
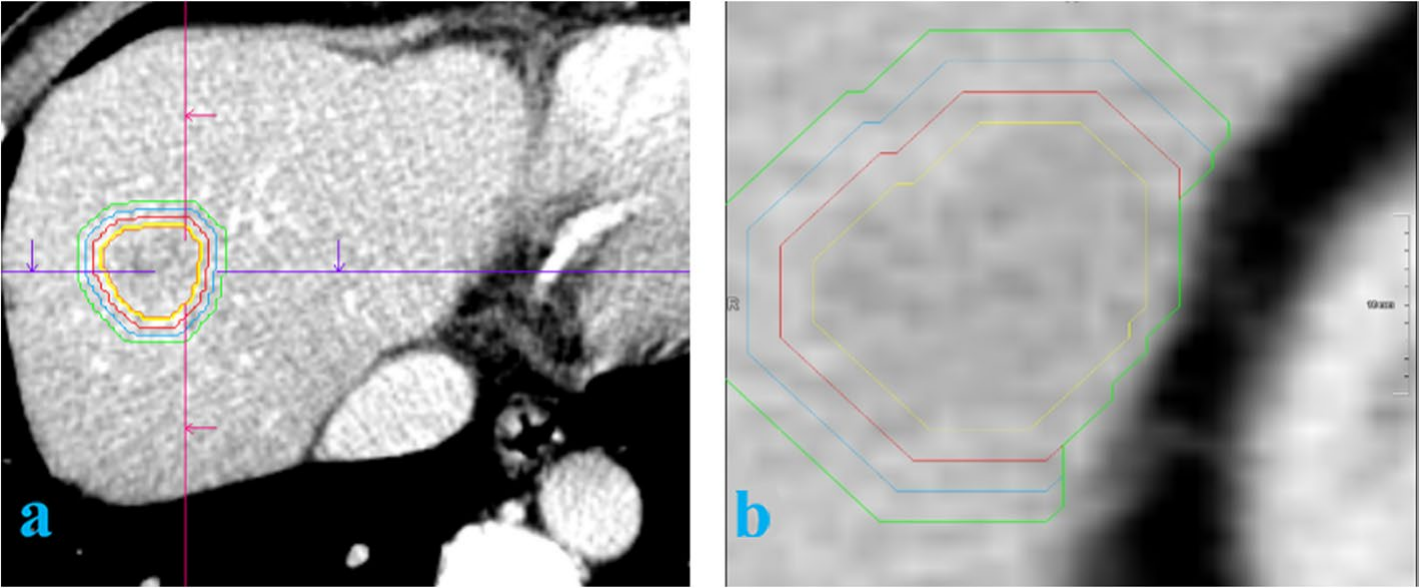
VOI was delineated and feature extraction: **a** and **b** represent those two patients and outline the volume process of interest. The yellow line represents the tumor boundary, the red line represents the physical expansion of 5 mm to the peritumor, the blue line represents the expansion of 10 mm, and the green line represents the expansion of 15 mm. In this study, 5-mm and 10-mm peritumor areas are discussed. **b** shows that peritumoral expansion terminates when it reaches the edge of the liver

The reconstructed images (in DICOM format) were fed into the radiomics software platform for VOI determination. The tumors were outlined using a semi-automatic general segmentation method (Fig. 2). Each of the two radiologists confirmed the region of interest within the specified range, making manual adjustments as needed to acquire the tumor VOI (Vtumor). After confirming the Vtumor, an automatic expansion algorithm expanded the area in all three dimensions. The peritumoral contour was automatically obtained. Care was taken to avoid blood vessels, gas, bones, and bile ducts, resulting in nonuniform Vtumor and Vperitumoral. The acquired VOIs from the arterial and portal vein phases were matched. To assess the consistency of VOI delineation, the process was repeated for 50 randomly selected patients at 1-month intervals. Intra-observer and inter-observer correlation coefficients (ICC) were strong (ICC > 0.80).

#### Feature extraction and model building

Once the VOIs were delineated, the features were computed using radiomics software. All features from the original radiomics software were used in the calculations. Consequently, each patient possessed six groups of features related to the arterial and portal vein phases, along with the peritumoral area. Each group included 1691 parameters, encompassing morphological, first-order, texture, filter transform, and wavelet transform features. Therefore, prioritizing dimensionality reduction screening was the primary approach when constructing a model.

First, stability feature extraction was conducted by evaluating features from 50 randomly chosen cases to determine inter-group and intra-group correlation coefficients. High-stability features (correlation coefficients > 0.80) were retained. Second, we used the Spearman correlation test to remove features with correlations. Third, hypothesis testing was performed. We used *T*-tests or Mann–Whitney rank sum tests to pick the features correlating with MVI. Fourth, we established the model using LASSO, which imposes constraints to reduce data dimensionality. This algorithm compresses coefficients associated with less influence to zero through a penalty function, ensuring that the sum of absolute values of the coefficients is less than a constant. Post-LASSO, robustness characteristics were obtained, generating an equation inclusive of weighted coefficients. The value determined by this equation was the Radscore.

### Statistical analysis

We used the R software package (version 3.6.1, http://www.R-project.org) to conduct the statistical analysis. Clinical baseline data were analyzed, and both univariate and multivariate logistic regression analyses were employed to identify potential risk factors associated with MVI. We used the Delong test to compare different models’ ability to predict MVI in HCC patients. *p* < 0.05 was deemed statistically significant. We used a decision curve to compare the models’ clinical value. Finally, we constructed a calibration curve to evaluate the model’s calibration capability.

## Results

### Patient characteristics

We included 206 eligible HCC patients, including 177 men and 29 women, among whom 61 were MVI-positive and 145 were MVI-negative. Out of these, 138 patients were randomly allocated to the training group and 68 to the validation group. The baseline patient data are presented in Table 1. All clinical indicators of the randomly grouped patients were statistically compared, and no statistical differences were observed across any indicators between the training and verification groups.

**Table 1:** Baseline data of 206 hepatocellular carcinoma patients

Character	Training (*n* = 138)	Validation (*n* = 68)	External validation (*n* = 60)	*H*/*χ*^2^ value	*p* value
Age (years)					
Median (IQR)	54.00 (48.00, 60.00)	56 (50.00, 61.75)	55 (52.00, 62.00)	-1.581	0.134
Range	19.00–72.00	31.00–81.00	26.00–72.00		
Gender					
Male	118	59	52	0.082	0.960
Female	20	9	8		
AFP (ng/mL)					
< 20	57	24	23	0.710	0.701
≥ 20	81	44	37		
Hepatitis B virus					
Yes	113	62	53	3.646	0.162
No	25	6	7		
Hepatitis C virus					
Yes	5	3	3	0.469	0.850
No	133	64	57		
Liver cirrhosis					
Yes	97	50	45	0.545	0.762
No	41	18	15		
Buga					
Yes	5	4	3	0.830	0.734
No	133	64	57		
Preoperative ALT (U/L)					
≥ 40	50	20	26	2.681	0.262
< 40	88	48	34		
Preoperative AST (U/L)					
≥ 40	51	19	26	3.367	0.186
< 40	87	49	34		
Tumor size					
≤ 2 cm	23	14	9	0.775	0.679
> 2 cm	115	54	51		
Child–Pugh score					
A	127	61	56	0.587	0.746
B–C	11	7	4		
Hepatic encephalopathy					
Yes	0	0	0		
No	138	68	60		
Ascitic fluid					
Yes	11	3	6	1.516	0.469
No	127	65	54		
Bilirubin (μmol/L)					
< 25	121	59	51	0.264	0.877
≥ 25	17	9	9		
Albumin (μmol/L)					
35–55	103	57	49	2.724	0.256
< 35 or > 55	35	11	11		
Prothrombin time prolonged					
4–6	0	0	0		
> 6	138	68	60		
Complete capsule					
Complete	73	33	26	2.036	0.729
Incomplete	52	29	29		
No	13	6	5		
Peritumoral enhanced					
Yes	24	10	14	1.687	0.430
No	114	58	46		
Smooth edge					
Yes	72	38	31	0.307	0.858
No	66	30	29		
Intratumoral arteries					
Negative	88	49	35	2.728	0.256
Positive	50	19	25		
Peritumoral low density					
Negative	101	54	46	1.007	0.605
Positive	37	14	14		
TTPVI					
Negative	103	52	42	0.744	0.689
Positive	35	16	18		
Tumor-liver difference					
Clear	107	45	40	4.100	0.129
Unclear	31	23	20		
RVI					
Negative	125	61	52	0.685	0.710
Positive	13	7	8		

### Univariate and multivariate analyses of clinical and imaging features

The study population was divided based on postoperative pathological MVI status, resulting in 61 MVI-positive patients and 145 MVI-negative patients. The probability of MVI was 29.6% (61/206). We conducted univariate and multivariate logistic regression analyses on all baseline data, including imaging features and pathological findings (Table 2). There was a significant correlation between capsular invasion and MVI. Elevated preoperative alanine aminotransferase (ALT) and aspartate transaminase (AST) levels were also associated with MVI. Factors such as peritumoral enhancement, tumor margin, existence of intratumoral arteries along with a low-density rim surrounding the tumor, integrity of the tumor capsule, and density differences between the tumor and the liver interface were all identified as MVI risk factors. The RVI and TTPVI mentioned in previous studies were also significantly correlated with MVI, indicating that they are risk factors for MVI. Multivariate logistic regression analysis confirmed that capsular invasion was an independent risk factor for MVI. TTPVI, peritumoral enhancement, capsule integrity, and density differences between the tumor and liver interface were also identified as independent MVI risk factors.

**Table 2:** Two hundred six patients’ univariate and multivariate logistic regression analysis

Variant	Univariate		Multivariate	
	OR (95%CI)	*p* value	OR (95%CI)	*p* value
**Gender**				
Male vs female	0.337 (0.112, 1.014)	0.053		
**Age**				
≧ 60 vs < 60 years	1.287 (0.680, 2.438)	0.438		
**AFP (ng/mL)**				
< 20 vs ≧ 20	1.586 (0.866, 2.906)	0.135		
**Pathology grade**				
I–II vs III–IV	1.810 (0.919, 3.563)	0.086		
**Capsular invasion**				
No vs yes	5.673 (2.020, 15.934)	0.001*	6.658 (2.054, 21.578)	0.002*
**Tumor size**				
≦ 2 cm vs > 2 cm	1.656 (0.710, 3.865)	0.243		
**Hepatitis B**				
No vs yes	0.616 (0.278, 1.363)	0.232		
**Hepatitis C**				
No vs yes	0.998 (0.630, 1.581)	0.994		
**Cirrhosis**				
No vs yes	0.546 (0.288, 1.035)	0.064		
**Budd-Chiari syndrome**				
No vs yes	3.147 (0.815, 12.150)	0.096		
**Preoperative ALT**				
≦ 40 U/L vs > 40 U/L	1.950 (1.044, 3.642)	0.036*		
**Preoperative AST**				
≦ 40 U/L vs > 40 U/L	1.884 (1.015, 3.496)	0.045*		
**Child–Pugh score**				
A vs B–C	0.657 (0.207, 2.082)	0.475		
**Hepatic encephalopathy**				
No vs yes				
**Ascitic fluid**				
No vs yes	1.868 (0.620, 5.634)	0.267		
**Total protein**				
≦ 25 vs > 25 μmol/L	1.907 (0.820, 4.433)	0.134		
**Albumin**				
35–55 μmol/L vs < 35 or 55 μmol/L	1.199 (0.592, 2.427)	0.614		
**Peritumoral enhanced**				
Yes vs no	6.252 (2.841, 13.759)	0.000*	3.911 (1.583, 9.664)	0.003*
S**mooth edge**				
Yes vs no	0.217 (0.113, 0.416)	0.000*		
**Complete capsule**				
Yes vs incomplete and no	0.217 (0.112, 0.421)	0.000*	0.447 (0.206, 0.972)	0.042*
**Intratumoral arteries**				
Negative vs positive	3.221 (1.722, 6.024)	0.000*		
**Peritumoral low density**				
Negative vs positive	0.192 (0.072, 0.512)	0.001*		
**Tumor-liver difference**				
Clear vs unclear	0.230 (0.119, 0.446)	0.000*	0.306 (0.142, 0.659)	0.002*
**TTPVI**				
Negative vs positive	5.067 (2.575, 9.971)	0.000*	2.368 (1.074, 5.223)	0.033*
**RVI**				
Negative vs positive	9.130 (3.146, 26.500)	0.000*		

*RVI* radiogenomic venous invasion, *TTPVI* two-trait predictor of venous invasion

^*^p < 0.05

### Selection and modeling of radiomics features

We excluded features with correlation coefficients > 0.90. Hypothesis testing was performed on the remaining radiomics features to select those with significant statistical differences between the groups characterized by the presence or absence of MVI. Finally, we employed the LASSO algorithm to identify and select the most predictive features for building the model.

The number of parameters of the 5-mm peritumoral radiomics models in the arterial phase is 21. Radscore A (V_per5mm_) = 0.29714285714285715

 − 0.074353 X_wavelet-HHL_glcm_Imc2
 − 0.057030 X_original_shape_Elongation
 − 0.053594 X_original_glcm_MaximumProbability
 − 0.049559 X_wavelet-LHH_glcm_ClusterShade
 − 0.037649 X_square_ngtdm_Busyness
 − 0.037427 X_exponential_glszm_SmallAreaEmphasis
 − 0.020075 X_original_glszm_SmallAreaLowGrayLevelEmphasis
 − 0.019563 X_wavelet-HLH_glcm_InverseVariance
 − 0.016828 X_logarithm_glcm_Imc1
 − 0.014204 X_log-sigma-0–5-mm-3D_firstorder_Median
 − 0.009435 X_original_glcm_Imc1
-  + 0.000737 X_square_glszm_LowGrayLevelZoneEmphasis
-  + 0.002325 X_original_glcm_Imc2
-  + 0.004567 X_wavelet-LHL_firstorder_Kurtosis
-  + 0.006704 X__wavelet-LLL_glcm_Correlation
-  + 0.008527 X_exponential_glcm_MCC
-  + 0.017787 X_log-sigma-4–5-mm-3D_gldm_SmallDependenceEmphasis
-  + 0.018892 X_wavelet-HLL_glcm_Correlation
-  + 0.041008 X_original_glcm_Correlation
-  + 0.041567 X_squareroot_firstorder_Kurtosis

-  + 0.049917 X_wavelet-LLL_glszm_GrayLevelNonUniformityNormalized

The number of parameters of the 10-mm peritumoral radiomics models in the arterial phase is 12. Radscore A (V_per10mm_) = 0.2894528153595409

 − 0.028023 X_square_ngtdm_Busyness
 − 0.019626 X_wavelet-HLH_firstorder_Mean
 − 0.013075 X_original_glcm_MaximumProbability
-  + 0.001592 X_original_glcm_Correlation
-  + 0.005310 X_exponential_glcm_Idmn
-  + 0.011947 X_exponential_glcm_Correlation
-  + 0.014276 X_original_shape_Maximum3DDiameter
-  + 0.017465 X_log-sigma-2–5-mm-3D_gldm_SmallDependenceEmphasis
-  + 0.024618 X_squareroot_glcm_Imc2
-  + 0.033227 X_original_glcm_Imc2
-  + 0.035365 X_original_gldm_DependenceEntropy
-  + 0.046991 X_wavelet-LHL_firstorder_Kurtosis

The number of parameters of the 5-mm peritumoral radiomics models in the portal venous phase is 14. Radscore V (V_per5mm_) = 0.2971428571428572

 − 0.036178 X_wavelet-LLL_ngtdm_Busyness
 − 0.030546 X_original_shape_Elongation
 − 0.020929 X_wavelet-HHL_glcm_Imc2
 − 0.020450 X_squareroot_glcm_Imc1
 − 0.014857 X_wavelet-LLL_glszm_SmallAreaLowGrayLevelEmphasis
 − 0.014509 X_wavelet-HLL_firstorder_Median
 − 0.009716 X_wavelet-LHH_glcm_InverseVariance
-  + 0.009029 X_original_shape_Maximum3DDiameter
-  + 0.012752 X_log-sigma-4–5-mm-3D_firstorder_90Percentile
-  + 0.019192 X_wavelet-HLL_glrlm_RunVariance
-  + 0.022603 X_squareroot_glcm_Imc2
-  + 0.027566 X_wavelet-LHH_glcm_Imc1
-  + 0.042216 X_log-sigma-0–5-mm-3D_ngtdm_Busyness
-  + 0.046826 X_wavelet-LLH_firstorder_InterquartileRange

The number of parameters of the 10-mm peritumoral radiomics models in the portal venous phase is 25. Radscore V (V_per10mm_) = 0.29714285714285743

 − 0.071165 X_wavelet-HHH_glcm_InverseVariance
 − 0.064444 X_wavelet-HHH_glszm_SizeZoneNonUniformityNormalized
 − 0.046372 X_original_glszm_ZoneEntropy
 − 0.037803 X_wavelet-LLL_ngtdm_Busyness
 − 0.034973 X_logarithm_gldm_SmallDependenceLowGrayLevelEmphasis
 − 0.033692 X_wavelet-LHL_gldm_SmallDependenceLowGrayLevelEmphasis
 − 0.031802 X_original_glcm_JointEnergy
 − 0.028619 X_log-sigma-1–5-mm-3D_firstorder_Median
 − 0.025689 X_wavelet-LLH_glrlm_RunEntropy
 − 0.016416 X_squareroot_glszm_ZoneEntropy
 − 0.014365 X_wavelet-LLL_glcm_JointEnergy
 − 0.012163 X_squareroot_glrlm_ShortRunLowGrayLevelEmphasis
 − 0.007045 X_original_ngtdm_Busyness
-  + 0.002252 X_original_gldm_DependenceEntropy
-  + 0.007024 X_log-sigma-1–5-mm-3D_glszm_GrayLevelNonUniformity
-  + 0.011931 X_logarithm_firstorder_RobustMeanAbsoluteDeviation
-  + 0.013431 X_logarithm_glszm_GrayLevelNonUniformityNormalized
-  + 0.023832 X_log-sigma-0–5-mm-3D_gldm_SmallDependenceLowGrayLevelEmphasis
-  + 0.028906 X_original_shape_Maximum3DDiameter
-  + 0.030032 X_logarithm_glcm_MCC
-  + 0.033813 X_log-sigma-4–5-mm-3D_firstorder_90Percentile
-  + 0.035153 X_wavelet-LLH_firstorder_RobustMeanAbsoluteDeviation
-  + 0.047734 X_original_firstorder_InterquartileRange
-  + 0.066691 X_wavelet-LLL_glcm_Imc2
-  + 0.113210 X_wavelet-HHL_glrlm_LongRunLowGrayLevelEmphasis

**Fig. 3:**
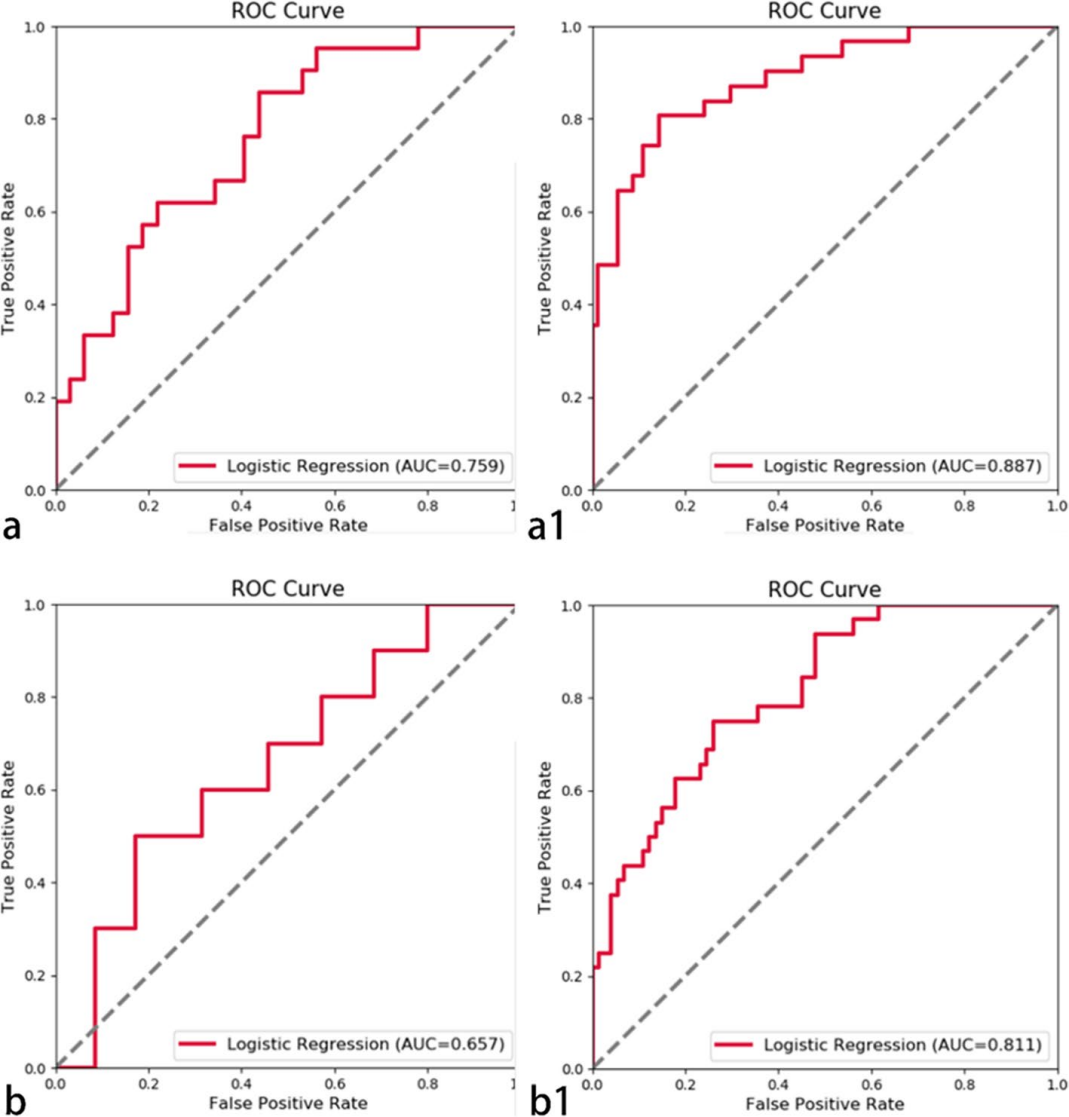
In the arterial phase, the AUC value of the peritumoral 5-mm (**a**) validation set was 0.759 and (**a1**) training set was 0.887. The AUC value of the 10-mm peritumoral (**b**) validation set was 0.657 and (**b1**) training set was 0.811

**Fig. 4:**
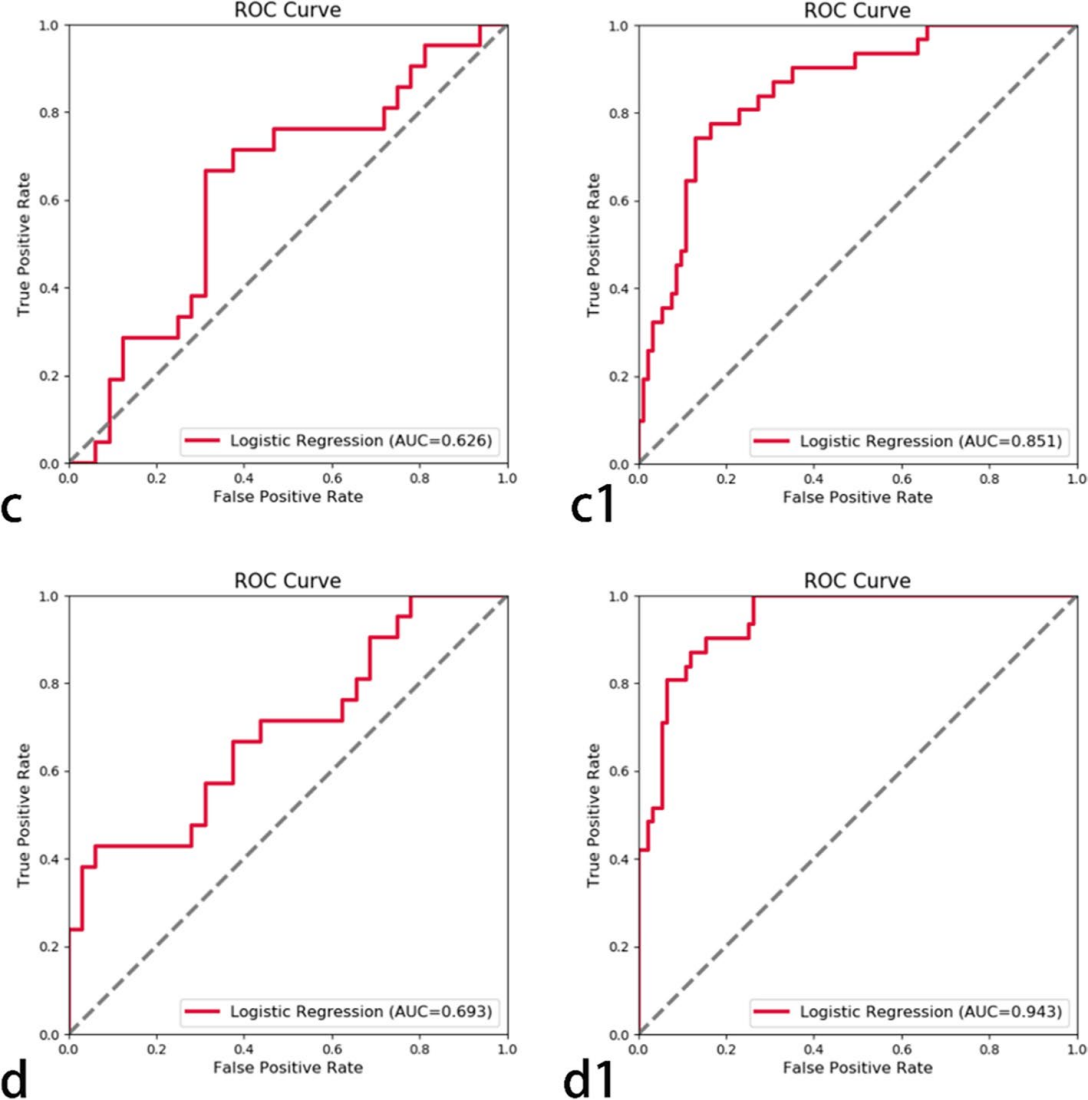
In the portal vein phase, the AUC value of the 5-mm peritumor (**c**) validation set was 0.626 and that of the (**c1**) training set was 0.851. The AUC value of the 10-mm peritumor (**d**) validation set was 0.693, and the AUC value of the (**d1**) training set was 0.943

The ROC illustrating model effectiveness is shown in Figs. 3 and 4. In the arterial phase, the 5-mm peritumor validation set AUC value was 0.759 and that of the training set was 0.887. Comparatively, for the 10-mm peritumoral validation set, it was 0.657, and for the training set, it was 0.811. In the portal venous phase, the AUC value of the 5-mm verification set was 0.626, and that of the training set was 0.851. The verification and training sets had AUC values of 0.693 and 0.943, respectively. The model validation results indicated higher diagnostic efficiency of the 5-mm peritumoral area in the arterial phase compared to the 10-mm peritumoral area, while the diagnostic efficiency of the 5- and 10-mm peritumoral areas in the portal vein phase showed similar diagnostic efficiency in the validation set. Therefore, the 5-mm peritumoral area is more efficient than the 10-mm area for predicting MVI.

In clinical practice, the diagnostic process often combines multi-stage imaging features. Therefore, this study combined the radiomics features of these four independent volumes of interest in pairs to generate combined models. The models including peritumoral radiomics features showed superior predictive efficacy compared to the models including tumors alone, and the arterial phase provided more diagnostic information to predict the MVI of HCC compared to the portal vein phase. The AUC values of the combined model’s training set were stable (> 0.85). The highest AUC value of the combined model’s test set was 0.82, which was defined as the optimal combined model. The model was established by combining the tumor and the 5-mm peritumoral imaging features in the arterial phase. Finally, 28 parameters were selected, 13 from tumor and 15 from 5-mm peritumoral imaging.

Radscore A(Vt) + A(Vper) = 0.29714285714285704

 − 0.061300 X(A)_original_glcm_MaximumProbability
 − 0.056516 X(A)_original_shape_Elongation
 − 0.045483 X(A)_wavelet-LHH_glcm_ClusterShade
 − 0.043287 X(A)_square_ngtdm_Busyness
 − 0.035068 X(A)_exponential_glszm_SmallAreaEmphasis
 − 0.034264 X(Aper)_wavelet-HHL_glcm_MCC
 − 0.028729 X(Aper)_wavelet-HLL_glrlm_ShortRunEmphasis
 − 0.024973 X(Aper)_wavelet-HHH_glcm_Contrast
 − 0.022994 X(Aper)_wavelet-LLL_firstorder_Range
 − 0.016860 X(A)_wavelet-HHL_glcm_Imc2
 − 0.014491 X(Aper)_wavelet-HHH_gldm_LowGrayLevelEmphasis
 − 0.011767 X(A)_original_glszm_SmallAreaLowGrayLevelEmphasis
 − 0.006108 X(Aper)_wavelet-LLL_firstorder_10Percentile
 − 0.005779 X(Aper)_logarithm_glcm_Imc1
 − 0.003976 X(A)_log-sigma-0–5-mm-3D_firstorder_Median
 − 0.002726 X(Aper)_wavelet-HHL_glcm_Imc2
 − 0.002492 X(Aper)_original_glszm_LowGrayLevelZoneEmphasis
-  + 0.005516 X(Aper)_log-sigma-2–5-mm-3D_gldm_SmallDependenceEmphasis
-  + 0.014060 X(A)_original_glcm_Correlation
-  + 0.021479 X(Aper)_logarithm_firstorder_Kurtosis
-  + 0.025073 X(A)_wavelet-LLL_glcm_Correlation
-  + 0.025199 X(Aper)_log-sigma-4–5-mm-3D_firstorder_90Percentile
-  + 0.030258 X(Aper)_logarithm_gldm_SmallDependenceHighGrayLevelEmphasis
-  + 0.030717 X(A)_wavelet-LLL_glszm_GrayLevelNonUniformityNormalized
-  + 0.031095 X(A)_wavelet-HLL_glcm_Correlation
-  + 0.044388 X(A)_squareroot_firstorder_Kurtosis
-  + 0.045011 X(Aper)_wavelet-LHL_glszm_SizeZoneNonUniformityNormalized
-  + 0.051918 X(Aper)_square_firstorder_Skewness

### Comparison between the optimal radiomics and clinical models

**Fig. 5:**
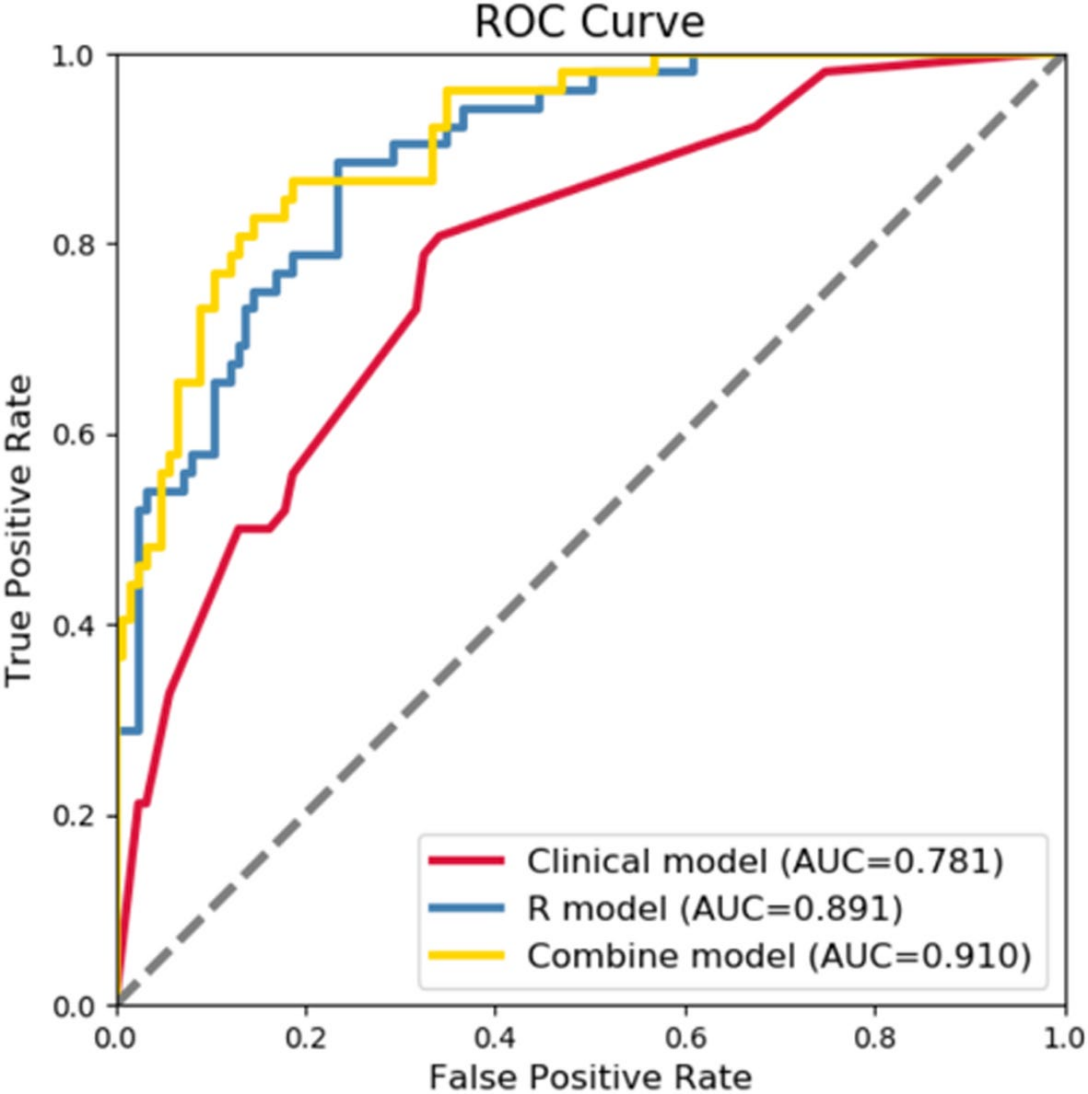
Comparison of the three models for predicting MVI. The AUC of the traditional clinical model (red line) was 0.781. The AUC of the optimal imaging combination model (blue line) was 0.891, and that of the clinical and omics fusion model (yellow line) was 0.910

After the multivariate logistic analysis, preoperative indicators were selected from the clinical baseline data to establish a preoperative diagnostic model of MVI based on traditional clinical data. The indicators included peritumoral enhancement, capsule status, density differences between the tumor and liver interface, and TTPVI. The fusion model was then established by combining optimal radiomics and clinical data. The comparative efficiencies of the three models are shown in Fig. 5. In terms of diagnostic efficiency, the optimal radiomics model had greater diagnostic efficiency than the traditional clinical model, and the fusion model had the highest diagnostic efficiency.

### Decision curve drawing

**Fig. 6:**
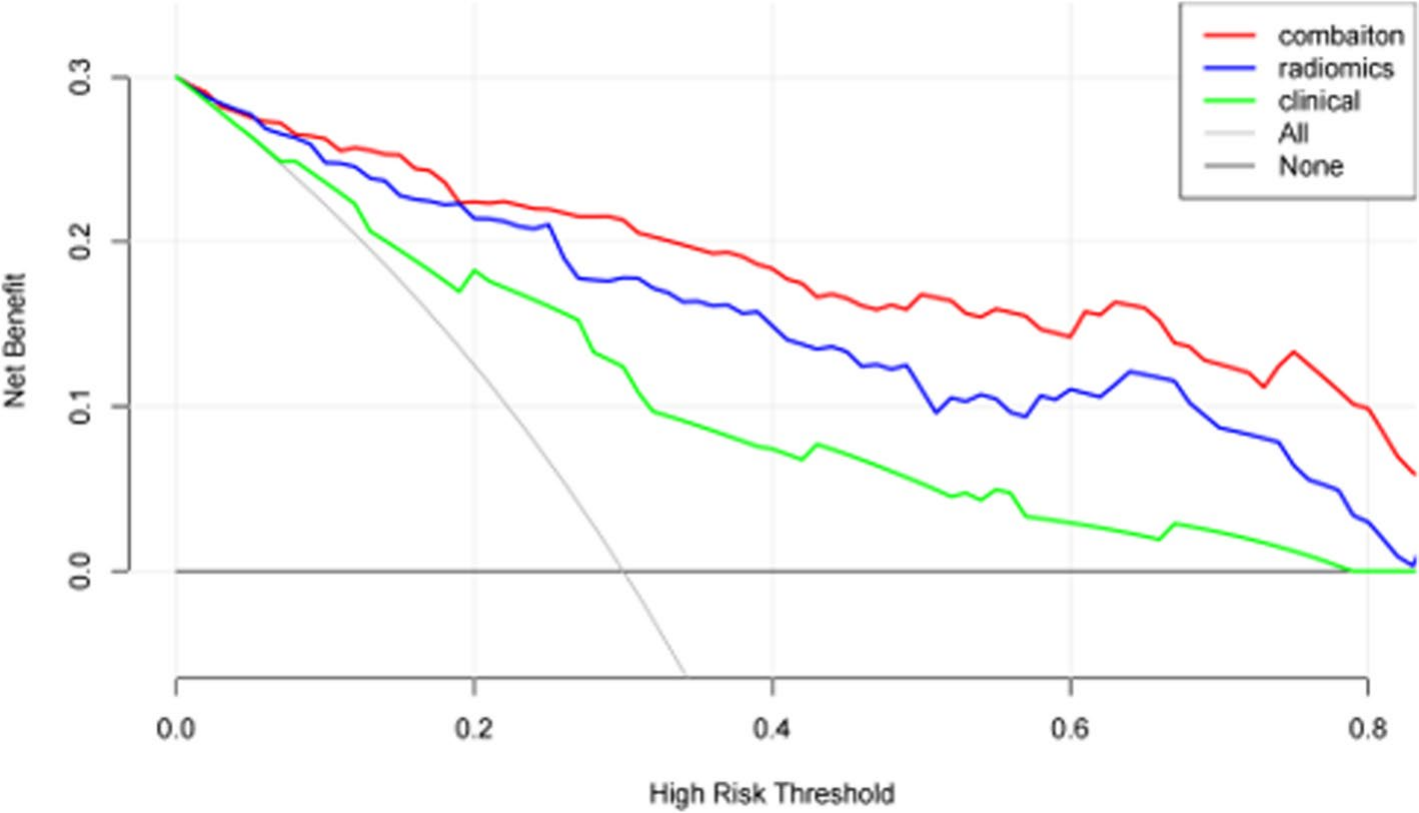
Decision curves of different models. The green line represents the clinical model, the blue line represents the radiomics model (arterial tumor + arterial peritumor 5 mm), and the red line represents the fusion model. From the analysis of the decision curve, it can be concluded that when the threshold probability range is greater than 0.1, the fusion model can obtain a large net benefit

The light gray diagonal line in Fig. 6 indicates the relationship between all models and the incidence of MVI across all patients. When the threshold probability exceeds 0.1, the benefits of the fusion model are greater than those of the single model, and the benefits derived from the tumor and peritumoral radiomics model surpass those of the traditional clinical model indicating that, in clinical practice, the benefits of the tumor and peritumoral radiomics model are slightly greater than those of traditional models, while the benefits of the fusion model are greater than any single model. The fusion model has good application value in clinical practice. In the external validation cohort, the optimal combination model exhibited a diagnostic AUC value of 0.643, while the AUC value of the clinical and radiomics fusion model reached 0.753.

## Discussion

Partial hepatectomy or liver transplantation is often more beneficial for patients with stage T2 tumors (early-stage HCC) than for patients in later disease stages. However, within the subset of early-stage HCC tumors measuring ≤ 5 cm, postoperative prognoses can vary significantly, with worse prognoses for patients with MVI. Therefore, this study focused on accurately predicting preoperative MVI statuses in patients with early-stage HCC in order to formulate individualized treatment plans for this specific patient group. Discussions on this tumor subgroup, particularly concerning CT-based radiomics, are rarely reported.

Regarding the biological behavior of HCC, many scholars have established MVI models to study the radiomics features of tumors through preoperative imaging [19]. Radiomics aims to uncover the biological nature of tumors using high-throughput data. Chen et al. conducted an MRI radiomics study of hepatobiliary-specific contrast agents, showing that the inclusion of radiomics features within a 1-cm peritumoral area could effectively predict intratumor immune score grading [20]. Similarly, an MRI-enhanced radiomics study of breast cancer showed that the combined radiomics features of the tumor and peritumoral area could effectively predict the efficacy of neoadjuvant chemotherapy [21]. A growing body of research indicates that small changes in the structure of normal tissues surrounding tumors can provide information regarding biological tumor behavior. A study of non-small cell lung adenocarcinoma showed that the 3-mm peritumoral region was different from the 3- to 9-mm peritumoral region and that the inclusion of the 3-mm peritumoral region significantly enhanced the radiomics model’s efficacy in predicting distant metastasis [22]. The abovementioned studies indicate that the peritumoral area can be used to obtain biological information about tumors; however, a unified definition of the peritumoral scope remains absent.

The results of this study showed that for HCC less than 5 cm, both the 5- and 10-mm peritumoral histological models held predictive value for MVI. ROC analysis of the model validation set revealed that the 5-mm peritumoral arterial histological model outperformed the 10-mm peritumoral model in diagnostic efficiency. No significant difference in diagnostic efficacy between the 5-mm peritumoral histological model and the 10-mm peritumoral model was noted at the portal vein stage. The diagnostic efficiency of the 5-mm peritumoral model in the arterial stage was the highest. Therefore, in the following discussion, a 5-mm peritumoral range is regarded as the focus of this study. In a separate study of lung adenocarcinoma, researchers found that including peritumoral data enhanced the diagnostic efficiency in distinguishing between benign and malignant nodules, but as the spread distance increased, the efficacy of the peritumoral diagnostic model gradually decreased [23], a trend that aligns with our study’s findings.

The combination of the tumor and peritumoral area in the arterial stage had the greatest diagnostic efficiency, with a training set AUC value of 0.941 and a validation set AUC value of 0.820. This model was determined to be the optimal combination model. MVI can change the hemodynamics around the tumor. Therefore, peritumoral models of the arterial and portal phases provide valuable information, and peritumoral diagnosis of the arterial phase was of greater significance in this study. A fusion model, including the optimal radiomics combination model and clinical indicators after multi-factor regression analysis, was established. Considering the wide application of preoperative enhanced CT in clinical practice, this study incorporated traditional imaging features into clinical data. Peritumoral enhancement is significantly correlated with MVI, which may be caused by changes in peritumoral blood flow and abnormal perfusion [24, 25]. The presence of intratumoral arteries often indicates rapid growth and strong invasiveness of tumors [26, 27]. Capsule integrity acts as a physical barrier to the tumor and can hinder the occurrence of MVI [28]. In this study, when the density difference between the tumor and liver interface was not significant, the incidence of MVI was higher, which is something that is not discussed in many studies. Here, we included the RVI and TTPVI in the clinical model, which has rarely been done by other researchers. Radiomics is a means of presenting the microscopic environment, which is otherwise invisible to the naked eye, in order to improve diagnostic accuracy. The fusion model established in this study also demonstrated optimal clinical application value in the decision curve analysis.

There are several limitations to this study. First, its retrospective nature comes with an inherent bias. While external validation was part of the experimental design, larger cohort sizes and additional external validations are necessary. Second, only 206 patients were included, most of whom had hepatitis-associated HCC. Future studies should also consider alcoholic liver disease or non-alcoholic steatohepatitis. Third, we did not examine the correlation between genomic and radiomics features, which could further clarify the impact of radiomics. Finally, our research, which was based on enhanced CT images, lacks multimodal image data.

## Conclusion

In conclusion, a radiomics model including a 5-mm peritumoral expansion stands as a promising noninvasive biomarker for preoperatively predicting MVI in individuals with a solitary HCC ≤ 5 cm. This model can aid in shaping personalized treatment policies.

## Data Availability

Please contact the author for data requests.

## Abbreviations

ALT: Alanine aminotransferase
AST: Aspartate transaminase
AUC: Area under the curve
CT: Computed tomography
HCC: Hepatocellular carcinoma
ICC: Intra-observer and inter-observer correlation coefficients
LASSO: Least absolute shrinkage and selection operator
MRI: Magnetic resonance imaging
MVI: Microvascular invasion
ROC: Receiver operating characteristic
RVI: Radiogenomic venous invasion
TTPVI: Two-trait predictor of venous invasion
VOI: Volume area of interest
Vtumor: Tumor VOI

## References

[1] Ferlay J, Colombet M, Soerjomataram I et al (2021) Cancer statistics for the year 2020: an overview. Int J Cancer. 10.1002/ijc.33588 10.1002/ijc.33588 33818764

[2] Raoul JL, Edeline J (2020). Apr Systemic treatment of hepatocellular carcinoma: standard of care in China and elsewhere. Lancet Oncol.

[3] Siegel RL, Miller KD, Fuchs HE, Jemal A (2021). Jan Cancer statistics, 2021. CA Cancer J Clin.

[4] Cheng Z, Yang P, Qu S (2015). Risk factors and management for early and late intrahepatic recurrence of solitary hepatocellular carcinoma after curative resection. HPB (Oxford).

[5] Ueno S, Kubo F, Sakoda M, Hiwatashi K, Tateno T, Mataki Y (2008). Efficacy of anatomic resection vs nonanatomic resection for small nodular hepatocellular carcinoma based on gross classification. J Hepatobiliary Pancreat Surg.

[6] Guo D, Gu D, Wang H, Wei J, Wang Z, Hao X (2019). Aug Radiomics analysis enables recurrence prediction for hepatocellular carcinoma after liver transplantation. Eur J Radiol.

[7] Zhang X, Li C, Wen T, Yan L, Li B, Yang J (2015). Aug Appropriate treatment strategies for intrahepatic recurrence after curative resection of hepatocellular carcinoma initially within the Milan criteria: according to the recurrence pattern. Eur J Gastroenterol Hepatol.

[8] Yao Z, Dong Y, Wu G, Zhang Q, Yang D, Yu JH, Wang WP (2018). Preoperative diagnosis and prediction of hepatocellular carcinoma: radiomics analysis based on multi-modal ultrasound images. BMC Cancer.

[9] Rodríguez-Perálvarez M, Luong TV, Andreana L, Meyer T, Dhillon AP, Burroughs AK (2013). Jan A systematic review of microvascular invasion in hepatocellular carcinoma: diagnostic and prognostic variability. Ann Surg Oncol.

[10] Lei Z, Li J, Wu D, Xia Y, Wang Q, Si A (2016). Nomogram for preoperative estimation of microvascular invasion risk in hepatitis B virus-related hepatocellular carcinoma within the Milan criteria. JAMA Surg..

[11] Chou CT, Chen RC, Lin WC, Ko CJ, Chen CB, Chen YL (2014). Sep Prediction of microvascular invasion of hepatocellular carcinoma: preoperative CT and histopathologic correlation. AJR Am J Roentgenol.

[12] Lambin P, Rios-Velazquez E, Leijenaar R, Carvalho S, van Stiphout RG, Granton P (2012). Mar Radiomics: extracting more information from medical images using advanced feature analysis. Eur J Cancer.

[13] Li H, Zhu Y, Burnside ES, Huang E, Drukker K, Hoadley KA (2016). Quantitative MRI radiomics in the prediction of molecular classifications of breast cancer subtypes in the TCGA/TCIA data set. NPJ Breast Cancer.

[14] Wu M, Tan H, Gao F, Hai J, Ning P, Chen J (2019). Jun Predicting the grade of hepatocellular carcinoma based on non-contrast-enhanced MRI radiomics signature. Eur Radiol.

[15] Yang G, Nie P, Zhao L, Guo J, Xue W, Yan L (2020). Aug 2D and 3D texture analysis to predict lymphovascular invasion in lung adenocarcinoma. Eur J Radiol.

[16] Yang Y, Fan W, Gu T, Yu L, Chen H, Lv Y (2021). Radiomic features of multi-ROI and multi-phase MRI for the prediction of microvascular invasion in solitary hepatocellular carcinoma. Front Oncol.

[17] Banerjee S, Wang DS, Kim HJ, Sirlin CB, Chan MG, Korn RL, Rutman AM, Siripongsakun S, Lu D, Imanbayev G, Kuo MD (2015). A computed tomography radiogenomic biomarker predicts microvascular invasion and clinical outcomes in hepatocellular carcinoma. Hepatology.

[18] Renzulli M, Brocchi S, Cucchetti A, Mazzotti F, Mosconi C, Sportoletti C (2016). May Can current preoperative imaging be used to detect microvascular invasion of hepatocellular carcinoma?. Radiology.

[19] Ma X, Wei J, Gu D, Zhu Y, Feng B, Liang M (2019). Jul Preoperative radiomics nomogram for microvascular invasion prediction in hepatocellular carcinoma using contrast-enhanced CT. Eur Radiol.

[20] Chen S, Feng S, Wei J, Liu F, Li B, Li X (2019). Aug Pretreatment prediction of immunoscore in hepatocellular cancer: a radiomics-based clinical model based on Gd-EOB-DTPA-enhanced MRI imaging. Eur Radiol.

[21] Braman NM, Etesami M, Prasanna P et al (2017) Intratumoral and peritumoral radiomics for the pretreatment prediction of pathological complete response to neoadjuvant chemotherapy based on breast DCE-MRI. Breast Cancer Res 19. 10.1186/s13058-017-0846-1 10.1186/s13058-017-0846-1 PMC5437672 28521821

[22] Dou TH, Coroller TP, van Griethuysen JJM, Mak RH, Aerts HJWL (2018). Peritumoral radiomics features predict distant metastasis in locally advanced NSCLC. PLoS One.

[23] Akinci D’Antonoli T, Farchione A, Lenkowicz J, Chiappetta M, Cicchetti G, Martino A (2020). Apr CT radiomics signature of tumor and peritumoral lung parenchyma to predict nonsmall cell lung cancer postsurgical recurrence risk. Acad Radiol.

[24] Wu S, Zhang N, Wu Z, Ren J, Can EL (2022). Feb Can Peritumoral Radiomics Improve the Prediction of Malignancy of Solid Pulmonary Nodule Smaller Than 2 cm?. Acad Radiol.

[25] Xu QQ, Shan WL, Zhu Y, Huang CC, Bao SY, Guo LL (2021). Jun Prediction efficacy of feature classification of solitary pulmonary nodules based on CT radiomics. Eur J Radiol.

[26] Marquardt JU, Galle PR, Teufel A (2012). Jan Molecular diagnosis and therapy of hepatocellular carcinoma (HCC): an emerging field for advanced technologies. J Hepatol.

[27] Qian X, Lu X, Ma X, Zhang Y, Zhou C, Wang F (2022). A multi-parametric radiomics nomogram for preoperative prediction of microvascular invasion status in intrahepatic cholangiocarcinoma. Front Oncol.

[28] He M, Zhang P, Ma X, He B, Fang C, Jia F (2020). Radiomic feature-based predictive model for microvascular invasion in patients with hepatocellular carcinoma. Front Oncol.

